# 69766 Bariatric surgery to achieve transplant in end-stage organ diseasepatients: A systematic review and meta-analysis

**DOI:** 10.1017/cts.2021.587

**Published:** 2021-03-30

**Authors:** Babak Orandi, Joshua Purvis, Robert Cannon, Blair Smith, Cora Lewis, Norah Terrault, Jayme Locke

**Affiliations:** 1 UAB; 2 USC

## Abstract

ABSTRACT IMPACT: Many who suffer from end-stage organ disease do not qualify for solid organ transplantation because of obesity; however, bariatric surgery offers the potential to render select patients transplant-eligible, and in some cases, may lead to weight loss that is sufficient to reverse end-stage organ disease. OBJECTIVES/GOALS: As obesity prevalence grows, more end-stage organ disease patients will be precluded from transplant. Numerous reports suggest bariatric surgery in end-stage organ disease may help patients achieve weight loss sufficient for transplant listing, though the published data are limited. METHODS/STUDY POPULATION: We performed a systematic review/meta-analysis of studies of bariatric surgery to achieve solid organ transplant listing. RESULTS/ANTICIPATED RESULTS: Among 82 heart failure patients, 40.2% lost sufficient weight for listing, 29.3% were transplanted, and 8.5% had sufficient improvement with weight loss they no longer required transplantation. Among 28 end-stage lung disease patients, 28.6% lost sufficient weight for listing, 7.1% were transplanted, and 14.3% had sufficient improvement following weight loss they no longer required transplant. Among 41 cirrhosis patients, 58.5% lost sufficient weight for listing, 41.5% were transplanted, and 21.9% had sufficient improvement following weight loss they no longer required transplant. Among 288 end-stage/chronic kidney disease patients, 50.3% lost sufficient weight for listing and 29.5% were transplanted. DISCUSSION/SIGNIFICANCE OF FINDINGS: Small sample size and publication bias are limitations; however, bariatric surgery may benefit select end-stage organ disease patients with obesity that precludes transplant candidacy.

